# Critical dynamics of endogenous fluctuations predict cognitive flexibility in the Go/NoGo task

**DOI:** 10.1038/s41598-017-02750-9

**Published:** 2017-06-06

**Authors:** Jaana Simola, Alexander Zhigalov, Isabel Morales-Muñoz, J. Matias Palva, Satu Palva

**Affiliations:** Helsinki Institute for Lifesciences, Neuroscience Center, University of Helsinki, P.O. Box 56 (Viikinkaari 4), FI-00014 Helsinki, Finland

## Abstract

Fluctuations with power-law scaling and long-range temporal correlations (LRTCs) are characteristic to human psychophysical performance. Systems operating in a critical state exhibit such LRTCs, but phenomenologically similar fluctuations and LRTCs may also be caused by slow decay of the system’s memory without the system being critical. Theoretically, criticality endows the system with the greatest representational capacity and flexibility in state transitions. Without criticality, however, slowly decaying system memory would predict inflexibility. We addressed these contrasting predictions of the ‘criticality’ and ‘long-memory’ candidate mechanisms of human behavioral LRTCs by using a Go/NoGo task wherein the commission errors constitute a measure of cognitive flexibility. Response time (RT) fluctuations in this task exhibited power-law frequency scaling, autocorrelations, and LRTCs. We show here that the LRTC scaling exponents, quantifying the strength of long-range correlations, were negatively correlated with the commission error rates. Strong LRTCs hence parallel optimal cognitive flexibility and, in line with the criticality hypothesis, indicate a functionally advantageous state. This conclusion was corroborated by a positive correlation between the LRTC scaling exponents and executive functions measured with the Rey-Osterrieth Complex Figure test. Our results hence support the notion that LRTCs arise from critical dynamics that is functionally significant for human cognitive performance.

## Introduction

Human psychophysical performance fluctuates in time scales from seconds to tens or hundreds of seconds so that similar behavioral outcomes are much more likely to appear in clusters than expected by chance^1–4^. Such spontaneous performance fluctuations have been observed in hit-rate (HR) and response time (RT) data measured in continuous performance tasks (CPTs), in which both the production of actions^2, 5^ and perceptual performance^3, 5, 6^ resemble 1/*f* noise. More specifically, trial-to-trial performance fluctuations exhibit power-law scaling behavior and are governed by long-range-temporal correlations (LRTCs).

Scale-free dynamics have been found to characterize also neuronal activity fluctuations^4, 7–13^ so that the neuronal scaling-laws are predictive of those in behavior^7, 14, 15^. Power-law scaling and LRTCs are suggestive of the underlying neuronal systems operating in or near a critical state^6, 16–19^. Such scale-free dynamics, however, could be explained also without criticality^20–23^ by the system having a slowly decaying memory and the past neuronal dynamics influencing the future with a long-range memory based continuity.

Operating near criticality endows the system with the greatest dynamic range, optimal representational capacity, and importantly, flexibility in reconfiguration among possible states^12, 17, 24, 25^. Hence, if the observed behavioral LRTCs and scale-free dynamics are caused by the underlying neuronal systems operating in a critical state, LRTCs should be positively correlated with flexibility of the system because the underlying metastability should allow for rapid transitioning between states^19^. Although neuronal scaling-laws have been shown to be predictive of behavioral scaling laws^7, 14, 15^, there is yet no direct evidence for that LRTCs and scale-free dynamics would be advantageous for flexibility in human cognitive performance.

In contrast, scale-free dynamics can also arise from a persistent, slowly decaying dependence of the current dynamics on the dynamics in the past^20–23, 26^. This ‘long-memory’ hypothesis would predict LRTCs to be negatively correlated with dynamic flexibility because the greater the system’s dependency of its past is, the more difficult it is to undergo flexible reconfigurations. This prediction is in line with previous work, which has shown that compared with resting state, the temporal memory of fMRI brain signals decreases during task performance and thereby putatively increases information processing efficiency through reduction of temporal redundancy^23^. As indirect evidence for this notion, signal variance is decreased during task performance^27^ as well as during aging^28, 29^. The relevance of the signal variability has also been evidenced in neuronal networks learning Boolean rules in multiple-task learning^30^. On the other hand, in the criticality context, the attenuation of LRTCs during sensory stimulation has been attributed to disruption of the endogenous critical dynamics^4^.

We aimed to test whether behavioral LRTCs were positively or negatively correlated with cognitive flexibility in order to test whether ‘criticality’ or ‘long-range memory’ is the more likely mechanism underlying the behavioral dynamics^26, 31, 32^. We used a simple Go/NoGo task to measure the dynamics of response time (RT) fluctuations and the errors in the task to quantify cognitive flexibility in transitioning between sustained attention and response inhibition^33^. In this task, the responses to the NoGo stimuli constitute *commission errors* that reflect unsuccessful response inhibition and thereby define a measure of cognitive flexibility that is ontologically independent of the RT dynamics.

The criticality and long-memory hypotheses yield dichotomous predictions about the relationship of LRTCs in RT fluctuations and cognitive flexibility. The criticality hypothesis predicts that if the RT LRTCs are behavioral-level manifestations of critical dynamics in the task-relevant brain systems^7^, the participants with the *strongest* correlations, *i.e*., the greatest LRTC scaling exponents, would operate closest to the critical point^34^ and would thus have the greatest flexibility in stimulus-response reconfiguration. Thus, participants with the greatest LRTCs would be best in inhibiting the responses to NoGo stimuli and the population would exhibit a negative correlation of LRTCs with the commission errors. On the other hand, the long-memory hypothesis posits that if LRTCs are a result of slowly decaying system memory^20–23^, the participants with the *weakest* correlations (smallest LRTC exponents) would have the best information processing efficiency^23^ for alternating flexibly between the Go and NoGo choices, which at the group level would be evidenced by the LRTCs being positively correlated with the commission errors.

We first assessed whether RT fluctuations in the Go/NoGo task exhibited scale-free dynamics and then tested whether these dynamics predicted individual cognitive flexibility. Our results show that participants with the strongest LRTCs in their RT fluctuations make the least amount of commission errors. Negative correlation of LRTCs with commission errors hence indicates a positive correlation with cognitive flexibility. This conclusion was corroborated by a positive correlation between LRTCs and executive control measured with a neuropsychological test of executive functions. Our findings thus favor the hypothesis that LRTCs are caused the system being in a critical state over the idea that these LRTCs would reflect long-memory dynamics.

## Results

**Figure 1:**
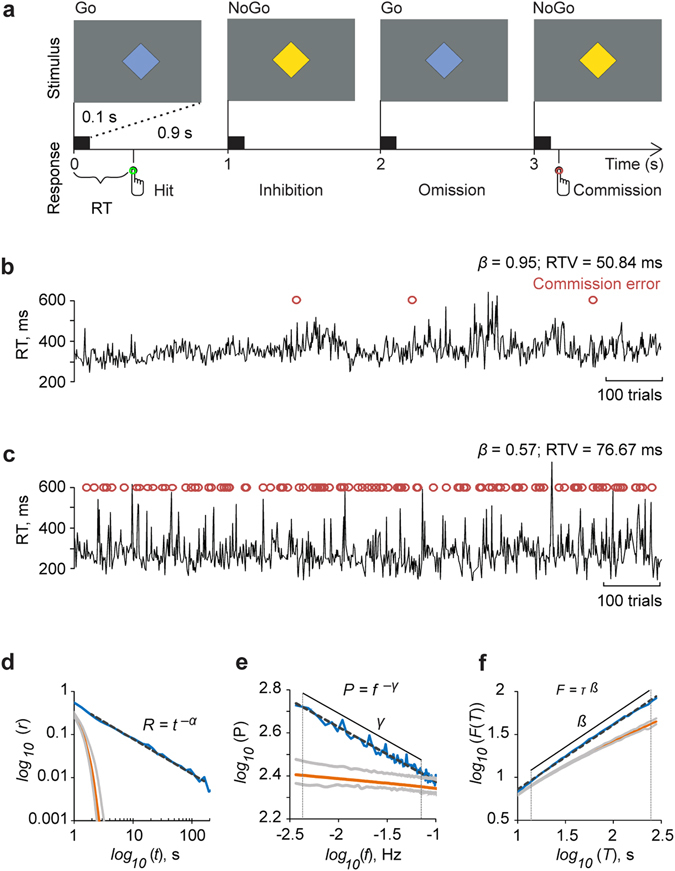
Scale-free dynamics characterizes response time (RT) fluctuations in the Go/NoGo task. (**a**) A schematics of the Go/NoGo task in which the blue diamond (1°) was the Go (75%) and the yellow (25%) the NoGo stimulus. The stimuli were presented for 100 ms followed by a fixed 900 ms inter-stimulus interval (ISI) during which a grey diamond was shown (not displayed here). Participants were instructed to respond to each Go stimuli and refrain from responding to the NoGo stimuli. Different responses are shown below the timeline. Responses to the Go stimuli were Hits and responses to NoGo stimuli commission errors. (**b**) The RT time-series collected from a good performer, *i.e*., one with few commission errors. This subject exhibited a large LRTC scaling exponent (*β*) that indicates strong power-law autocorrelations in the RT time series. (**c**) RT time-series of a poor performer, *i.e*., one with many commission errors. Here a low LRTC exponent value (*β*) indicates that temporal correlations in the RT time series are close to the ‘no-correlations’ level expected for white noise (*β* = 0.5). (**d**) The autocorrelation function (ACF) averaged across the participants (*n* = 27) and Go stimulus colors in double logarithmic coordinates shows a linear and thus power-law decay of the correlation coefficient (*r*) as a function of time lag (*t*) with the power-law scaling exponent *α* = 0.63 of the grand-average ACF. (**e**) The grand-average power spectral density (PSD) function shows a power-law decay of power with frequency with scaling exponent *γ* = 0.25. Vertical lines mark the frequency range 0.004–0.063 Hz used in the estimation of scaling exponent. (**f**) Grand-average detrended fluctuation analysis (DFA) wherein the fluctuation measure, F(*τ*), exhibits a power-law relationship with the window size, *τ*, in the *τ* range from 15 to 250 s and with the scaling exponent, *β* = 0.73. In panels e,-f, the corresponding analyses of surrogate data (see Methods) are plotted in orange with the grey lines indicating 99% confidence limits. The hand symbols in Fig. 1a are copyright free. The authors acknowledge IIT Bombay for the design.

A cohort of healthy volunteers (*n* = 27) completed two 17 min sessions of visual Go/NoGo tasks wherein they responded to frequently (75%) presented Go stimuli and withheld responses to less frequent (25%) NoGo stimuli (Fig. 1a). The sessions were separated by an approximately 1-hour break and the colors of Go and NoGo stimuli (blue or yellow) were switched between the sessions. The RT time-series exhibited complex dynamics with visually salient inter-trial as well as inter-individual variability (Fig. 1b,c).

### Behavioral RT dynamics in Go/NoGo task are scale-free and exhibit LRTCs

The dynamic nature of the RT fluctuations in Go/NoGo tasks has not been assessed earlier. We first asked whether these RT fluctuations would be characterized by power-law correlations as predicted by the hypothesized underlying critical dynamics. We used autocorrelation function (ACF) (Fig. 1d), power spectral density (PSD) (Fig. 1e), and detrended fluctuation analysis (DFA) (Fig. 1f) to first characterize the scaling properties of RTs time series. We found a decay in the ACF of RT time series to be well fit by a power law (*R* ^2^ = 0.95) and to remain above 99-% confidence limits of surrogate data for time lags up to ~100 s (Fig. 1d). This implies the presence of long-range correlations and slow components in these fluctuations, which was confirmed by PSD that was log-log linear and in the low-frequency end, clearly above the surrogate confidence limits up to 0.063 Hz (in time scales from at least 15 to 250 s, power-law fit *R* ^2^ = 0.97, Fig. 1e). Finally, the presence of slow fluctuations and power-law long-range temporal correlations (LRTCs) in RT time series was also observed with DFA (Fig. 1f) that is a robust indicator of scale-free dynamics. The DFA exponents, *β*, ranged from 0.54 to 0.95 with a mean of 0.73 ± 0.02 (± SD, average goodness of fit *r* ^*2*^ = 0.98 ± 0.08 SD) and were clearly above the *β* = 0.5 of white noise as well as the exponents of surrogate data (*β* = 0.56 ± 0.004, *r* ^*2*^ = 0.98 ± 0.05) (Table 1). These three lines of data together indicate the presence of power-law scaling behavior and LRTCs in the Go/NoGo task RT fluctuations. Importantly, for scale-invariant time series exhibiting genuine LRTCs, the ACF (*α*), PSD (*γ*), and DFA (*β*) scaling exponents are theoretically coupled^6, 35^ by the relationship *β* = (2 − *α*)/2 = (1 + *γ*)/2. We found this to hold well in our empirical data at the level of the mean exponents (observed mean *α = *0.63 ± 0.29 and mean *γ* = 0.26 ± 0.11, Table 1). We also found the correlations between *β* and (2 − *α*)/2 (*r* = 0.31, *p* = 0.024, Pearson correlation test, two-tailed) and between *β* and (1 + *γ*)/2 (*r* = 0.58, *p* < 0.0001) to be significant among the scaling exponents of individual subjects (see Table 1). These findings are thus compatible with the idea of the RT time series being generated by a critical system.

**Table 1:** Consistency of performance.

	Go stimulus color			
	Blue	Yellow	*t*(26)	*p*
α	0.65 (0.30)	0.61 (0.27)	0.51	0.614
α (*surrogate*)	1.35 (0.09)	1.34 (0.12)	0.61	0.549
(2 − *α*)/2^1^	0.67 (0.15)	0.70 (0.13)		
γ	0.25 (0.08)	0.26 (0.14)	0.25	0.802
γ (*surrogate*)	0.05 (0.04)	0.05 (0.04)	0.47	0.644
(1 + *γ*)/2^1^	0.62 (0.04)	0.63 (0.07)		
*β*	0.72 (0.09)	0.74 (0.10)	0.80	0.432
*β* (*surrogate*)	0.56 (0.02)	0.56 (0.02)	0.61	0.546
Omission errors, %	2.35 (4.21)	3.98 (8.58)	1.28	0.212
Commission errors, %	18.64 (11.39)	20.00 (9.61)	0.85	0.403
RTV, ms				
Go	67.67 (19.27)	70.40 (24.82)	0.75	0.458
NoGo	61.95 (31.17)	54.21 (25.16)	1.12	0.272
MRT, ms				
Go	316.86	321.09	0.71	0.483
NoGo	273.62	277.68	0.73	0.471

Mean (SD) of the ACF (*α*), PSD (*γ*) and DFA (*β*) scaling exponents and performance measures in the two versions of the Go/NoGo task, with either blue or yellow Gostimulus color. Omissions refer to the non-responded Gostimuli and commissions to responses to NoGostimuli. The *t* and *p* values are obtained from paired samples *t*-tests between the two versions of the task with switched stimulus colors.

^1^For time series originating from a critical system and exhibiting genuine LRTCs, the ACF (*α*), PSD (*γ*) and DFA (*β*) scaling exponents are theoretically coupled^6, 35^ by the relationship *β* = (2 − *α*)/2 = (1 + *γ*)/2.

### LRTCs of RT fluctuations are associated with cognitive flexibility in the Go/NoGo task

**Figure 2:**
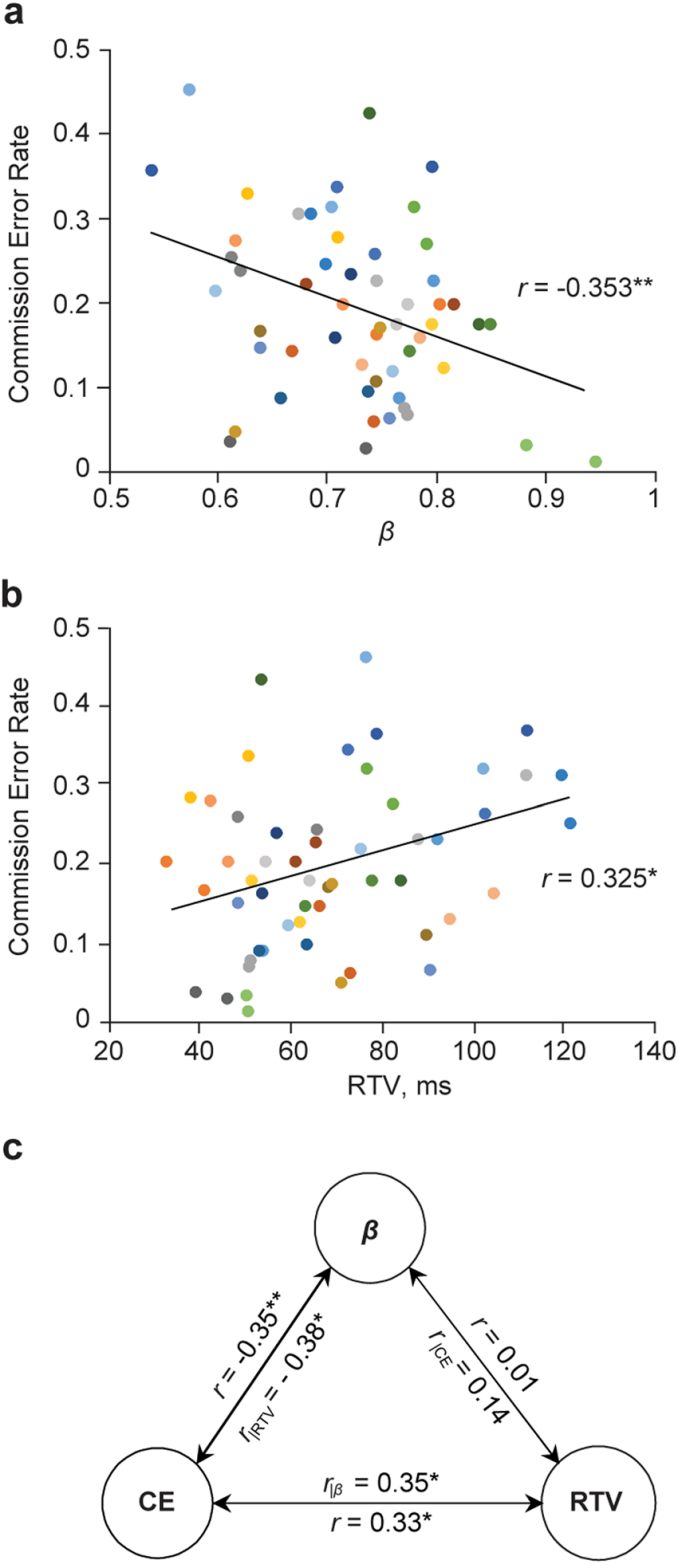
Response inhibition is correlated with long-range temporal correlations. (**a**) Correlation between the proportion of commission errors and the DFA scaling exponents (*β*). Each participant (*n* = 27) is indicated with colored dots twice, once for the blue and once for the yellow Go stimulus condition. The line shows the best-fit linear-regression line for the whole dataset. (**b**) The same for the correlation between commission error rates and response time variability (RTV, the SD of response times in milliseconds). (**c**) Correlations and partial correlations between the commission error rates (CE), DFA scaling exponents (*β*) and RTV. *p < 0.05, **p < 0.01.

To test the two alternative hypotheses, we asked whether the individual LRTC scaling exponents, *β*, were correlated with the primary outcome measure of the Go/NoGo task, the commission errors. All correlations are based on two-tailed Pearson correlation test. We found that the scaling exponents were negatively correlated with the fraction of commission errors (*r* = −0.35, *p* < 0.009; Fig. 2a). The participants with strongest correlations in their RT time series were thus most capable to inhibit responses to the NoGo stimuli (see Fig. 1b,c). The participants’ behavior was consistent and test-retest reliable in the two experimental sessions measured in terms of the scaling exponents, percentage of omission and commission errors, response time variability (RTV), and mean response time (MRT) (Table 1), which suggests that the Go/NoGo task performance in here taps into individual traits of the participants. The fraction of omission errors was low (Table 1) and uncorrelated with the LRTC scaling exponents, *β* (*r* = −0.088, *p* = 0.526).

### LRTCs in RT fluctuations are uncorrelated with response speed and variability

Response time variability (RTV), typically measured by the standard deviation of the RTs, is a key measure of behavioral dispersion. Higher RTV in tasks such as sustained attention to response task (SART)^36^, stop signal task (SST)^37^, and continuous performance test (CPT)^37^, as well as in Go/NoGo task^37^ characterize individuals with attention-deficit/hyperactivity disorder (ADHD) and may thus reflect compromised executive control^38^. We estimated the correlation between commission errors and RTV in our data, and in line with prior studies^39^, found that fraction of errors was indeed positively correlated with RTV (*r* = 0.33, *p* < 0.016; Fig. 2b). To disentangle the contributions of LRTCs and RTV on commission errors, we assessed their mutual correlation and performed a three-way partial correlation analysis. The LRTC scaling exponents were clearly uncorrelated with the RTV (*r* = 0.01, *n.s*.; Fig. 2c), while both the LRTCs and RTV were correlated with commission errors also in the partial correlation analysis (Fig. 2c). Furthermore, while the mean response time (MRT) was correlated with the fraction on commission errors (*r* = −0.34, *p* < 0.013) and with the RTV (*r* = 0.60, *p* < 0.00001), MRT was uncorrelated with the LRTC scaling exponents (*r* = 0.01, *n.s*.). The measures of response times and their variability (MRT and RTV) thus tap onto cognitive determinants of task performance that are distinct from those related to LRTCs and, putatively, critical brain dynamics.

### LRTCs in RT fluctuations are positively correlated with executive cognitive performance

**Figure 3:**
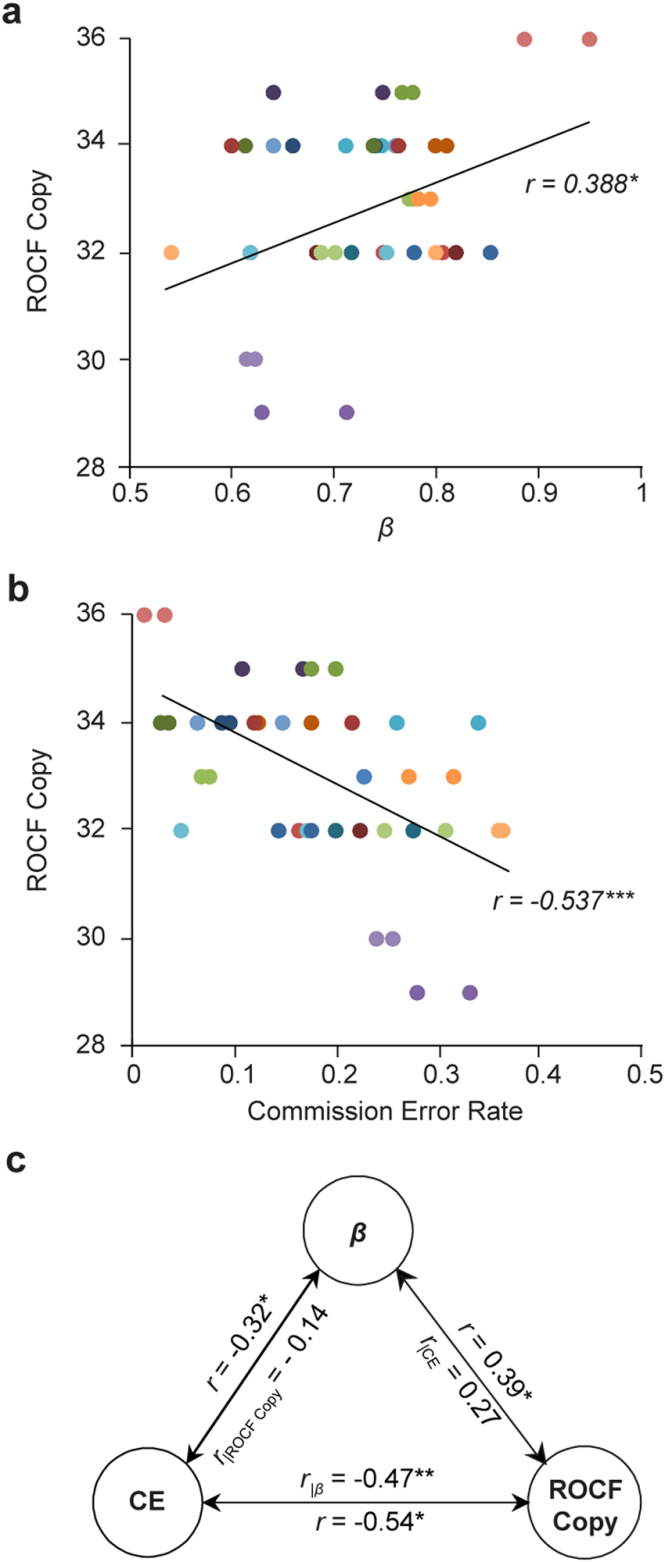
Long-range temporal correlations are predictive of individual variability in general executive functions. (**a**) Correlations between the accuracy score of copying the Rey-Osterrieth Complex Figure (ROCF Copy score; on the y-axis) and the DFA scaling exponents (*β*) (on the ﻿x﻿-axis). Each participant (*n* = 21) is indicated with colored dots twice, once for the blue and once for the yellow Gostimulus condition. The line shows the best-fit linear-regression line for the whole dataset. (**b**) Correlations between the ROCF Copy score and the proportion of commission errors. (**c**) Correlations and partial correlations between the commission error rates (CE), DFA scaling exponents (*β*) and the ROCF Copy scores. *p < 0.05, **p < 0.01, ***p < 0.001.

To next assess whether the relationship between scale-free dynamics and cognitive flexibility in the Go/NoGo task would generalize to another measure of cognitive flexibility and executive control, we used the well-established Rey-Osterrieth Complex Figure (ROCF) test. In the ROCF test, the participant copies an abstract design and the response is scored based on the organizational quality of the copied design. Due to the complexity of the figure, the ROCF test measures executive functions such as organizational and planning abilities^40, 41^. Furthermore, the ROCF performance is significantly correlated with performance in the Wisconsin Card Sorting Test (WCST) that also measures cognitive flexibility^41^. We first estimated the correlation of the LRTC scaling exponents with ROCF scores that ranged from 29 to 36 with an average score of 33 (±1.7 SD). The ROCF scores, and hence executive cognitive performance, were positively correlated with the LRTC scaling exponents (*r* = 0.39, *p* < 0.05; Fig. 3a). Moreover, we found a negative correlation between the ROCF scores and commission error rates in the Go/NoGo task (*r* = −0.54, *p* < 0.001; Fig. 3b), indicating that participants who made less commission errors had higher accuracy in the ROCF. The ROCF scores, on the other hand, were uncorrelated with RTV (*r* = 0.04, *n.s*.). Partial correlation analysis of LRTCs, ROCF, and commission errors showed that the ROCF scores were clearly correlated with commission errors (Fig. 3c) even when LRTCs were factored out. However, when the ROCF scores were controlled for, the correlation between LRTC scaling exponents and commission error rates was attenuated. This strongly suggests that the Go/NoGo commission errors and ROCF performance are based on partially shared underlying cognitive mechanisms that both are significantly coupled with individual scaling dynamics. Taken together, these results indicate that the scale-free behavioral dynamics is functionally significant in cognitive tasks and implies that high LRTCs gives an advantage in neuropsychological tasks requiring flexible executive cognitive control.

## Discussion

In this study, we addressed whether critical-state or long-memory dynamics was a more likely explanation for the 1/*f* -like fluctuations and LRTCs in human behavioral time series. To this end, we first characterized the scaling behavior of the RT fluctuations in Go/NoGo task. We found that these RT time series exhibited highly significant power-law auto correlations, frequency scaling, and LRTCs. These scaling properties were mutually consistent with a self-similar process, as expressed by the theoretical relationship proposed by^6, 35^, and thus provided an appropriate phenomenological foundation for testing the hypothesis that these fluctuations could arise from the system being at the critical state^7^. We contrasted this hypothesis with an alternative interpretation of these observations, namely that while there indeed are auto-correlated temporal structures in human behavior, these correlations reflect long-range memory dynamics where slow decay in the system’s memory govern the dynamics without criticality^20–23^.

Importantly, these two alternative hypotheses have dichotomous predictions for how 1/*f* fluctuations and LRTCs are correlated with cognitive flexibility, which we tested by quantifying the relationship between LRTC scaling exponents and cognitive flexibility measured by commission error rates in the Go/NoGo task. In the criticality framework, strong LRTCs are an indication of the system operating closer to the critical point than weak LRTCs. The criticality hypothesis thus predicts that subjects with stronger LRTCs operate close to the critical point and therefore have greater cognitive flexibility endowed by criticality through facilitation of meta-stable state transitions^12^ and optimal representational capacity^19^. Conversely, the long-memory model predicts that participants with stronger autocorrelations or LRTCs would have weaker cognitive flexibility because stronger correlations, *i.e*., greater power-law scaling exponents here, indicate that the past dynamics of the system have stronger influence on its future dynamics^23^. This in turn limits flexibility and possibilities for rapid reconfiguration similarly to how inertia limits the changes in motion of a physical object. Our data showed that the individual LRTCs of the RT fluctuations were negatively correlated with individual commission error rates. Participants operating closer to the critical point in terms of RT fluctuations thus appeared to have greater cognitive flexibility as indexed by their ability to switch responses between the Go and NoGo stimuli. These data thus clearly favor the hypothesis that critical-state dynamics underlies the LRTCs in human performance.

To corroborate this interpretation, we used another widely used measure of cognitive flexibility, the ROCF test. We found that, indeed, both the individual LRTCs and Go/NoGo commission error rates were related to central executive functions measured by the ROCF test that reflects organizational and planning abilities^40, 41^. That is, participants with greater scaling exponents were also more accurate in the ROCF test. Beyond addressing the two key questions, these results comprise an important novel demonstration of critical brain dynamics being functionally significant and useful for human cognitive performance. Our data suggest that criticality could be essential in allowing adaptive and flexible mental and behavioral dynamics.

The Go/NoGo task is one of the most commonly used cognitive paradigms in investigations of inhibitory control and cognitive flexibility. Commission errors and response time variability therein have been shown to reflect individual differences in such executive functioning and constitute an endophenotype in several neuropsychiatric diseases such as attention deficit disorder (ADHD)^39^. We found that the associations between LRTC scaling exponents and central executive functions were uncorrelated with both response speed and variability and thus reflected and independent underlying cognitive construct compared to criticality.

Both the present and prior^7, 42, 43^ data together suggest individual variation in scale-free behavior as a candidate endophenotype that could enhance the understanding of variation between health and various neuropsychological and psychiatric conditions. For example, assessing individual variation in scale-free behavioral dynamics may improve the utility of neuropsychological assessment in ADHD, which has so far provided inconsistent findings regarding the deficits of cognitive functions in ADHD^44^. Understanding the functional implications of neuronal criticality and its abnormalities could thus open new ways for the diagnostics and treatment of diseases of the central nervous system.

## Methods

### Participants

Twenty-seven right-handed participants (mean age: 30, range: 23–43, females: 15), reporting no history of neurological or psychological impairment, completed the Go/NoGo task. Twenty-one participants (mean age: 30, range: 24–43, females: 11) of the original sample completed the neuropsychological ROCF task. No prior estimates of effect size existed for the phenomenon studied here. Therefore, we pre-specified the sample size to be at least 20 participants, with each participant contributing two measurement points (*i.e*., one for each experimental session). Prior to the participation, an informed consent was obtained from all participants. The experiment was approved by an ethical committee of the Helsinki University Central Hospital and conducted in accordance with the ethical standards of the Declaration of Helsinki.

### Task design

Throughout the experiment, an empty diamond (1° from a fixed viewing distance of 57 cm) was presented in the middle of the screen (height: 30 cm, width: 53 cm, resolution: 1920 × 1080, 60 Hz refresh rate) on a gray background with the mean luminance of 65 cd/m^2^. On the onset of each trial, the diamond became filled with blue (122 cd/m^2^) or yellow (139 cd/m^2^) for 100 ms. Participants were instructed to respond with their right index finger as quickly as possible when a Go stimulus appeared (75% of trials) and withdraw from responding when a NoGo stimulus appeared (25% of trials). The Go and NoGo -stimuli were distributed randomly in the stimulus stream with a constant stimulus onset asynchrony (SOA) of 1 s (Fig. 1a). In order to evaluate the consistency of the obtained results, the trials were divided into two blocks consisting of 1000 trials each. The blocks were presented in separate sessions with an approximately 1-hour break in between. The Go and NoGo stimulus colors (blue or yellow) were switched between the sessions. The order of the presenting the Go stimulus colors was counterbalanced across participants, such that 13 participants began the task with the blue and 14 with the yellow Go stimuli. Stimulus timing was controlled by Presentation™ software (Neurobehavioral Systems, Inc., Albany, CA, USA).

### Data analysis

Correctly responded Go-trials (‘hits’) and incorrectly responded NoGo-trials (‘commission errors’) were both included into the RT time series for our analysis but so that extreme RT values (<150 ms and >800 ms) were excluded. To corroborate that the analyses were not biased by the NoGo trials, we performed the LRTC analyses (DFA, see below) for RT time series containing only ‘hit’ trials and found that the DFA scaling exponents (*β*) were effectively the same as with those estimated with all RTs (*r* = 0.979, Pearson correlation test, two-tailed). Therefore, all responses were included into the analyses. The time series were constructed by the RTs conceptually allocated to the onsets of the stimuli.

The temporal correlations of the RT fluctuations were quantified using three approaches: autocorrelation, power spectrum and detrended fluctuation analysis (DFA). The decay, *α*, of the autocorrelation function was estimated using power-law function, *R*_*t*_ = *t* ^−*α*^. The autocorrelation function was computed as,

$${{R}}_{{t}}=\frac{1}{(N-t){\sigma }^{2}}\sum _{n=1}^{N-t}({x}_{n}-\mu )({x}_{n+t}-\mu )$$

where *x* denotes RTs time series; µ and σ are the mean and variance of time series; *N* denotes number of samples; *t* is the time lag. The slope, *γ*, of power spectrum plotted in log-log coordinates was approximated by a linear function, log(*P*_*f*_) = *γ* log(*f*).

The power spectrum was estimated using discrete Fourier transform,

$${P}_{f}={|\sum _{n=0}^{N-1}{x}_{n}\cdot {e}^{2\pi if(n/N)}|}^{2}$$

where *x* denotes RTs time series; |⋅| is the absolute value operator; *N* denotes number of samples.

The temporal correlations of the RT fluctuations were quantified using detrended fluctuation analysis (DFA)^45^. The DFA was applied to the time series of the RTs for both stimulus colors separately. In the first stage of DFA, the time series were normalized to zero mean and a cumulative sum of the signal was computed $y(k)=\sum _{i=1}^{k}[x(i)-\langle x\rangle]$. The integrated time series were then segmented into multiple time windows Δ*t* from 30 to 300 seconds. In the second stage of DFA, each segment of integrated data was locally fitted to a linear function *y*_Δ*t*_ and the mean-squared residual *F*(Δ*t*) was computed,

$$F({\rm{\Delta }}t)=\sqrt{\frac{1}{N}\sum _{k=1}^{N}{[y(k)-{y}_{{\rm{\Delta }}t}(k)]}^{2}}$$

where *N* is the length of time series.

The power-law scaling exponent, *β*, is defined as the slope of linear regression of the function *F*(Δ*t*) plotted in double logarithmic coordinates. The scaling exponent *β* can be considered a measure of temporal clustering so that higher *β* values indicate stronger temporal dependencies while values closer to 0.5 are associated with uncorrelated noise.

The scaling parameters of processes with long-range temporal correlations (LRTCs) obey the following relationship^35^, *β* = (2 − α)/2 = (1 + γ)/2. We observed a fairly similar relationship between the exponents in our data, 0.73 ± 0.02 (s.e.m.) ≅ 0.68 ± 0.04 ≅ 0.63 ± 0.02 (see Table 1).

### Statistical analysis

The α, γ, and *β* values of individual participants were compared with the α_s_, γ_s_, and *β*_*s*_ for surrogate data that were generated by random shuffling of RTs. This data driven approach allows estimating the chance level for the scaling exponents without making assumption on distribution of data. The relationship between the individual LRTC scaling exponents, response time variability (RTV), and the accuracy of the Go/NoGo and ROCF task performance was measured by calculating Pearson correlation coefficients. The test-retest reliability of the RT dynamics across the two experimental sessions was assessed using two-tailed paired samples *t*-test for the following measures: ACF (*α*), PSD (*γ*), DFA (*β*) and the respective surrogate data. The Go/NoGo task performance between the two sessions was compared for (i) percentage of omission errors (non-responded Go trials), (ii) percentage of commission errors (responses to NoGo trials), (iii) response time variability (RTV), and (iv) the mean response time (MRT) for the Go stimuli and erroneously responded NoGo stimuli (Table 1).

### Neuropsychological test

The Rey-Osterrieth Complex Figure Test (ROCF) was administered according to the Boston Qualitative Scoring System (BQSS) involving a copy condition, and immediate recall and a 20–30 min delayed recall. The ROCF was drawn with four colored pens, which were changed in fixed order, to enable us to track the drawing sequence. The present analysis was restricted to the accuracy of the copy condition, because this phase most strongly reflects executive functions^40, 41^. The current analysis for the ROCF test included the accuracy and placement of copying each element in the figure, which generate a score according to a 36-point scoring system^46^. The relationship between executive cognitive control and LRTC scaling exponents as well as the accuracy of the Go/NoGo task performance was measured by calculating Pearson correlation coefficients between the ROCF test score, the LRTC scaling exponents and the commission error rates.
